# The *MFN2* V705I Variant Is Not a Disease-Causing Mutation: A Segregation Analysis in a CMT2 Family

**DOI:** 10.1155/2013/495873

**Published:** 2012-11-28

**Authors:** Obaid M. Albulym, Danqing Zhu, Stephen Reddel, Marina Kennerson, Garth Nicholson

**Affiliations:** ^1^Northcott Neuroscience Laboratory, ANZAC Research Institute, Concord, NSW 2139, Australia; ^2^Sydney Medical School, University of Sydney, Sydney, NSW 2008, Australia; ^3^Molecular Medicine Laboratory, Concord Hospital, Concord, NSW 2139, Australia

## Abstract

Charcot-Marie-Tooth (CMT) disease is a clinically and genetically heterogeneous group of disorders affecting both motor and sensory neurons in the peripheral nervous system. Mutations in the *MFN2* gene cause an axonal form of CMT, CMT2A. The V705I variant in *MFN2* has been previously reported as a disease-causing mutation in families with CMT2. We identified an affected index patient from an Australian multigenerational family with the V705I variant. Segregation analysis showed that the V705I variant did not segregate with the disease phenotype and was present in control individuals with an allele frequency of 4.4%. We, therefore, propose that the V705I variant is a polymorphism and not a disease-causing mutation as previously reported.

## 1. Introduction

Charcot-Marie-Tooth (CMT), also known as hereditary motor and sensory neuropathy, is classified into two major categories: type 1 and type 2 based on the value of motor median nerve conduction velocity (NCV) [1]. CMT1, also known as the demyelinating form of CMT, is clinically characterized by slow NCVs due to myelin sheath abnormalities. CMT2 is as an axonal degeneration and is characterized by the reduction of the amplitudes of motor and sensory nerve action potentials with relatively normal conduction velocities [2]. The most common subtype of CMT2 is CMT2A, an autosomal dominant axonal degeneration of motor and sensory nerves caused by mutations in the mitochondrial mitofusin-2 (*MFN2*) gene (MIM 608507) [3]. Mutations in the *MFN2* gene are the most common cause for CMT2 [2–6]. *MFN2* is involved in mitochondrial fusion and the maintenance of mitochondrial morphology [7–9]. Numerous studies have reported mutations in the *MFN2* gene; the majority are point mutations [10]. We have identified the single base change c.2113G > A changing valine to isoleucine (V705I) of the *MFN2* gene in a patient from a multigenerational Australian family (CMT105) with CMT2 and pyramidal signs. The V705I variant of *MFN2* has been previously reported as a disease causing mutation in single individuals with CMT2 [11, 12]. To determine if this variant causes CMT2 in the family, we performed segregation analysis and tested the variant in a cohort of ethnically matched controls of English descent. 

## 2. Methods 

### 2.1. Subjects

**Figure 1:**
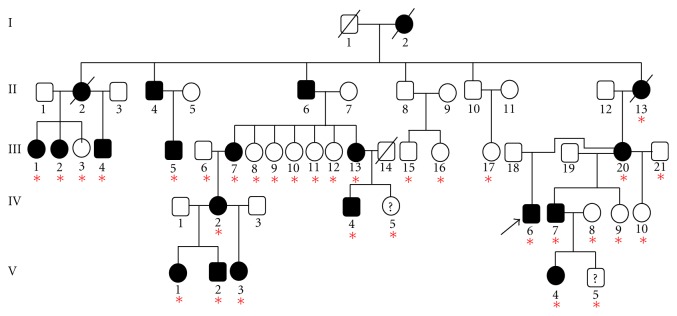
Pedigree of family CMT105. Circles and squares denote females and males, respectively. Open symbols indicate unaffected individuals and solid symbols indicate affected individuals. Symbols with diagonal lines denote deceased individuals. Symbols with question mark denote unknown phenotype. Asterisks denote family members recruited for the study. The V705I variant was initially detected in the index patient IV-6 (arrow).

Thirty-two individuals from a large, multigeneration Australian CMT2 family (CMT105) were tested (Figure 1). Sixteen individuals were clinically affected. Informed consent was obtained from all participants according to protocols approved by the Sydney Local Health District Human Ethics Committee. Genomic DNA was extracted from peripheral blood using standard methods by the Molecular Medicine Laboratory, Concord Hospital, Australia. 

### 2.2. High Resolution Melt (HRM) Analysis of *MFN2*

The coding regions of the *MFN2* gene were screened in the index patient IV-6 (Figure 1). The reference sequence for *MFN2* (NM_014874) was obtained from the National Centre for Biotechnology Information (http://www.ncbi.nlm.nih.gov/). Primer3 was used to design primers to amplify the coding exons and intron-exon boundaries of the *MFN2* gene. Primer sequences are available on request. DNA samples were amplified in a total volume of 10 *μ*L containing 10 ng genomic DNA, 1X HRM Master Mix (Idaho Technology), 4 pmol of each primer, and 1X PCR Enhancer (Invitrogen). A PCR Enhancer was not required to amplify exons 8, 9, 10, 13, and 16. DNA samples were amplified using the Eppendorf Mastercycler pro Thermal Cycler. PCR conditions were as follows: initial denaturation of 95°C for 15 min followed by 35 cycles of 95°C for 30 s, appropriate annealing temperature for 30 s, and 68°C for 40 s, with a final extension of 68°C for 5 min. Amplicons were scanned by high resolution melt (HRM) analysis on a 96-well Light Scanner (Idaho Technology). Melt curve profiles were analysed using Light Scanner Call-IT 2.0 software (Version 2.0.0.1331) as described previously [13]. Patient samples showing differential melt curve were sequenced using BigDye Terminator Cycle Sequencing at the Australian Cancer Research Foundation Facility, Garvan Institute of Medical Research, Australia. A sequence analysis was performed using SeqMan II version 5.03 (DNASTAR Inc.). 

### 2.3. Segregation Analysis

Exon 18 was screened through additional family members by HRM analysis. Thirty-two individuals from the family were tested for segregation and sixteen individuals were clinically affected with CMT2.

### 2.4. Control Testing

 One hundred and fourteen healthy chromosomes from 57 ethnically matched unrelated controls (English descent) were analysed for the presence of the V705I variant. Controls were nonconsanguineous spouses of other research subjects who had no signs of neuropathy. A PCR product containing V705I region was cut by *Aat* II restriction enzyme, which recognises GACGT. This site was erased by the V705I variant. A PCR amplicon was digested in a total volume of 20 *μ*L according to the conditions recommended by the manufacture. The products were separated on 2% of agarose gel.

## 3. Results

### 3.1. Clinical Findings

Detailed clinical features of this large Australian family with CMT2 and pyramidal signs have been previously published [14, 15]. The family pedigree (Figure 1) shows autosomal dominant inheritance with the disease phenotype segregating in five generations. Affected individuals in the family showed a reduction of compound amplitudes of motor and sensory nerve action potentials. Nerve conduction velocities were within the normal range. The disease was slowly progressive. Two individuals (IV-6 and III-2) were wheelchair bound with severe CMT2. Electrophysiological findings of these two patients are shown (Table 1). 

### 3.2. High Resolution Melt (HRM) Analysis

**Figure 2:**
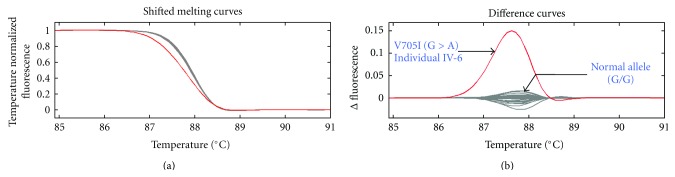
Subtractive fluorescent difference plots of affected and unaffected family members and unrelated controls for exon 18. Both healthy family members and affected individuals grouped with control individuals (grey). Patient IV-6 showed a different melt curve profile and formed a separate melt shape (red) reflecting the presence of the V705I variant.

**Figure 3:**
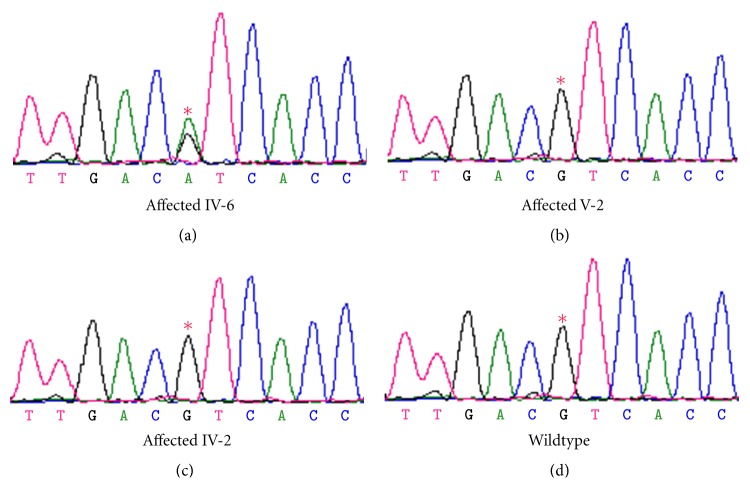
Sequence chromatograms showing the V705I variant in exon 18. An asterisk denotes the base change resulting in c.2113G > A (V705I). Panels (a), (b), and (c) are sequence chromatograms of clinically affected individuals. Panel (d) is the sequence chromatogram for the wildtype allele from a control individual. Only individual IV-6 carried the variant.

The *MFN2* gene was screened by HRM analysis in the index patient (IV-6) from CMT105 and a differential melt curve for exon 18 was observed when compared with the melt curves of control individuals (Figure 2). Dideoxy sequence analysis identified the following nucleotide transition c.2113G > A (V705I). We tested for segregation of the variant in the family. A unique differential melt curve corresponded to individual IV-6 was obtained while the melt curves of other affected individuals tightly grouped together with nonaffected family members and controls (Figure 2). This demonstrated that the V705I variant was not present in other affected family members and, therefore, the V705I was not segregating with the disease phenotype. To confirm the HRM findings, the index patient IV-6, two additional affected individuals, and a healthy individual from the family were sequenced. The analysis showed that the heterozygous V705I variant was present only in the index patient IV-6 while it was absent in other two affected individuals and the normal individual (Figure 3). No homozygous V705I variant was identified. We examined the frequency of the V705I in 57 healthy and ethnically matched controls of English descent and identified the heterozygous variant in five control samples (5/57) with an allele frequency of 4.4%.

## 4. Discussion

The *MFN2* V705I variant was found in an index patient (IV-6) from a large kindred with CMT2 and pyramidal signs. No other known or novel CMT mutations were found in the family. The V705I variant did not segregate with the disease in the family. The mother (III-20) and brother (IV-7) of the index patient are clinically affected but the variant was absent in both cases, indicating that the V705I variant was probably inherited from the father who was said to be unaffected and, unfortunately, was not available for testing. Furthermore, another individual (III-2) had similar clinical severity to the index patient (Table 1) and did not carry the V705I variant. This suggests that the V705I variant has no role in modifying the disease phenotype. 

To test the frequency of this variant in the population, we screened 57 unrelated controls (114 chromosomes) and detected the heterozygous V705I variant in 4.4% of these subjects. Our data, therefore, suggests that the variant, in its heterozygous state, is not a disease-causing mutation as previously reported [11]. 

Compound heterozygous mutations in *MFN2* have been identified in early onset CMT2 where *MFN2* mutations are not pathogenic unless coinherited with another *MFN2* mutation [16–18]. However, the *MFN2* gene was excluded in our family by linkage analysis [15]. Until a homozygous V705I variant is identified, the pathogenicity of the homozygous state will remain unknown and will require further studies if identified. 

The *MFN2* variant, V705I, was first reported as a pathogenic mutation in 2006 [11] in a proband clinically diagnosed with CMT2. This change leads to the exchange of the nonpolar valine to the nonpolar isoleucine at position 705 (c.2113G > A) (Figures 2 and 3). In the initial report, the variant was not detected in 212 ethnically matched control chromosomes from Norway. DNA samples from family members of the proband in the study were not available and therefore segregation of the variant has not been tested. The V705I variant has also been reported in two probands from unrelated Norwegian patients with CMT2 [12]. However, segregation of the variant in the families was not reported. 

Our thirty-two family members allowed further validation studies of the variant. The current dbSNP (Build 137, Jun 2012) database (http://www.ncbi.nlm.nih.gov/projects/SNP/) showed 690 SNP variants in the *MFN2* gene. The Inherited Peripheral Neuropathies Mutation database (http://www.molgen.ua.ac.be/CMTMutations/Home/Default.cfm) shows about 46 pathogenic mutations (6.3%). Therefore, any newly found variant is more likely to be a polymorphism than pathogenic mutation. 

Proof that a DNA variant is a disease-causing mutation requires rigorous validation when reporting novel sequence alterations [19–21]. This approach has been shown to be necessary in other diseases. The P1148A substitution in *FBN1* gene was initially thought to be a pathogenic mutation causing Marfan syndrome in the majority of patients of Asian descent [22]. This variant was subsequently described as a common polymorphism in Asian populations [23, 24]. 

We have now shown that the heterozygous *MFN2* V705I variant does not segregate with the disease proving that it is not a disease-causing mutation in this family. The identification of V705I in 5/57 ethnically matched normal controls establishes that in the heterozygote state this is not pathogenic. Our observation is supported by a recent report in dbSNP 135 (rs142271930), where the V705I variant was found in normal controls with minor allele frequency of 0.4% indicating that it is a rare variant in that population, whereas the frequency in the Australian population may be higher. Furthermore, our findings emphasise the importance of segregation studies and the use of many healthy controls from a variety of ethnic groups when describing novel, potentially pathogenic mutations. 

## 5. Conclusion

We have shown that the heterozygous *MFN2* V705I variant is a polymorphism and not a disease-causing mutation in our family. We have also previously excluded *MFN2* by linkage analysis. As this was previously reported as a disease-causing mutation, our study highlights the importance of variant validation by segregation studies and genotyping in ethnically matched controls. Further studies are needed to investigate the pathogenicity of homozygous *MFN2* V705I variant if identified.

## Figures and Tables

**Table 1:** Clinical motor and sensory electrophysiology study performed in the index patient IV-6 and individual III-2.

Patient	Age	Motor				Sensory			
		Median		Ulnar		Median		Ulnar	
		MAP	CV	MAP	CV	SAP	CV	SAP	CV
IV-6	41 y	1.5	49	1.2	59	0	NO	0	NO
III-2	66 y	—	56	—	58	0	0	0	0

MAP: motor action potential (mV), CV: conduction velocity (m/s), SAP: sensory action potential (*μ*V), NO: not obtainable, and (—): not done. Normal motor values were as follows: median MAP > 4.2 mV, median CV > 49 m/s, ulnar MAP > 5.6 mV, and ulnar CV > 47 m/s. Normal sensory values were as follows: median SAP > 9.0 *μ*V, median sensory CV > 56 m/s, ulnar SAP > 8 *μ*V, and ulnar sensory CV > 55 m/s.
